# COVID-19-related acute invasive fungal rhinosinusitis: risk factors associated with mortality

**DOI:** 10.1186/s43163-022-00332-9

**Published:** 2022-10-22

**Authors:** Kamal Ebied, Abraam Yacoub, Mohamed Gamea, Mohammad Salah Mahmoud

**Affiliations:** 1 Department of Otolaryngology-Head and Neck Surgery, Faculty of Medicine, Tanta University, Tanta, Egypt; 2 Department of Otolaryngology-Head and Neck Surgery, Faculty of Medicine, Ain Shams University, 38 Abbassia, Next to the Al-Nour Mosque, Cairo, Egypt; 3 Department of Otolaryngology-Head and Neck Surgery, Faculty of Medicine, Misr University for Science and Technology, October City, Egypt

**Keywords:** Acute invasive fungal sinusitis, Mucormycosis, COVID-19, Risk factors, Mortality, Survival, SARS-CoV-2

## Abstract

**Background:**

Acute invasive fungal rhinosinusitis (AIFRS) is a rare aggressive life-threatening infection that affects immunocompromised individuals. Recently, an increase in the incidence of this infection has been reported in patients who have SARS-CoV-2 infection or recently recovered. This study was to assess the outcome and define risk factors that might affect the outcome in SARS-CoV-2-related AIFRS. A prospective observational study included 54 patients diagnosed with SARS-CoV-2-related AIFRS. Controlling the predisposing factors, systemic antifungal, and early surgical debridement was performed. The mortality rate was calculated. Age, sex, underlying risk factors, the extent of the disease, debridement technique, and other biochemical variables were evaluated regarding their impact on survival. Patients were followed up for 3 months.

**Results:**

Fifty-four patients with a mean age of 48.1 years. Diabetes mellitus was the most common comorbidity affecting 52 patients (96.3%). Intracranial and intraorbital extension had a predictive value for mortality (*P* value 0.050 and 0.049 respectively). However, only intracranial extension was the independent predictor of mortality. Biochemical variables were higher than the normal range, but only serum ferritin level above 165 ng/ml was an independent predictor of mortality in patients with AIFR. The mortality rate was 38.9%.

**Conclusion:**

The extent of the disease has a major impact on survival, so early diagnosis of AIFRS within patients infected with SARS-CoV-2 or recently recovered is essential to reduce mortality.

## Background

Acute invasive fungal rhinosinusitis (AIFRS) is an uncommon life-threatening disease that can opportunistically infect immunocompromised patients with a weak neutrophilic response, such as those with blood malignancies, or uncontrolled diabetes mellitus (DM) [1]. It can also affect immunocompetent individuals with massive soft tissue injury or a state of iron overload [2]. AIFRS is caused by several filamentous fungi including Mucorales, Aspergillus, and Candida. These fungi are found *ubiquitously* in soil and decaying vegetation [3].

The mortality rate is determined by the underlying conditions as well as the extent of infection [4]. Early diagnosis, extensive surgical debridement along with systemic antifungal, and control of underlying risk factors are all part of the treatment strategy [5]. Without early detection and intervention, the disease can progress quickly, with mortality rates around 50–80% especially if extra sino-nasal extension occurred (orbital and intracranial complications) [6]. However, in some patients, even with early detection, control of underlying diseases, and vigorous medical and surgical intervention, management is often ineffective, leading to the dissemination of infection and, eventually, death [7].

Recently, the number of AIFRS cases has spiked with the consecutive waves of the SARS-CoV-2 pandemic [8–10]. It was suggested that co-infection or post-infection with SARS-CoV-2 elevates the risk of developing AIFR [11]. According to a recent study, 8% of coronavirus-positive or recovered patients developed secondary bacterial or fungal infections during the hospital stay, following substantial use of broad-spectrum antibiotics and steroids [12].

The immunosuppression induced by SARS-CoV-2 infection, or the intensive use of steroids and broad-spectrum antibiotics in the management of SARS-CoV-2 infection, can contribute to the development or exacerbation of a pre-existing fungal disease, or even changes in innate immunity associated with SARS-CoV-2 infection may be attributable to the reduced cluster of differentiation (CD) 4 and CD 8 T lymphocytes [13].

This study was conducted as factors associated with the poor outcome have not been thoroughly investigated in patients with AIFRS induced by SARS-CoV-2 infection. Hence, this study aimed to evaluate patient-related factors that might affect the survival of those patients.

## Methods

### Study design

This is a prospective cohort study that was conducted at a tertiary care referral center according to the international ethical standards and the Helsinki Declaration.

### Patient selection

After the institutional review board approval (Tanta University, Faculty of Medicine Ethics Committee), informed consent was obtained from the study population. Patients diagnosed with AIFRS after or during SARS-CoV-2 infection were recruited between January 2021 and March 2021. Fifty-four patients (24 females and 30 males) had the criteria of proven AIFRS [14, 15], with positive rt-PCR for SARS-CoV-2 infection and a disease course fewer than 4 weeks. Patients were excluded in any of the following conditions; no proven histopathology for AIFRS, disease course longer than 4 weeks, not proven SARS-CoV-2 infection by rt-PCR, or unknown outcome due to lost follow-up.

### Work-up

A detailed medical history was taken regarding any associated medical disease (hypertension, chronic kidney disease, hepatic diseases, hematologic malignancy, or diabetes mellitus). A further detailed history of diabetes mellitus was obtained (duration, medications, control, follow-up, and complications).

Head and neck examination was carried out including endoscopic nasal examination and ophthalmological examination; necrosis of the nasal turbinates or the septum, facial skin necrosis, visual acuity, and perception of light were documented. Oxygen saturation at the time of presentation was recorded and monitored.

All patients had computed tomography (CT) of the nose and paranasal sinuses to detect sinuses involvement, and bony erosions. Also, CT of the chest was performed to assess the degree of lung affection according to Chest CT Severity Score [16] and to rule out pulmonary mucormycosis. Magnetic resonance imaging (MRI) of the nose, paranasal sinuses, skull base, and brain was done if indicated to evaluate the extent of the disease (orbital involvement, intracranial extension, pterygopalatine fossa, and infratemporal fossa extension) [17].

In addition to the routine preoperative laboratory work-up, fasting and 2-h post-prandial blood glucose levels, glycosylated hemoglobin, serum ferritin, serum lactate dehydrogenase (LDH), and initial C-reactive protein (CRP) were done. Once AIFRS was suspected clinically, systemic antifungal (deoxycholate amphotericin B) was administered under the supervision of the infectious disease unit. Reversal of the underlying predisposing factor while preparing the patient to undergo surgical debridement if the general condition permits, usually within 48 h.

Surgical treatment was initially performed by the endoscopic endonasal approach. We debrided the necrosed tissues until macroscopically healthy tissue with bleeding edges was encountered, together with obtaining a non-necrosed tissue for histopathological examination. The extent of the disease could require combining open approaches (orbital exenteration and maxillectomy) with the endoscopic debridement.

**Fig. 1:**
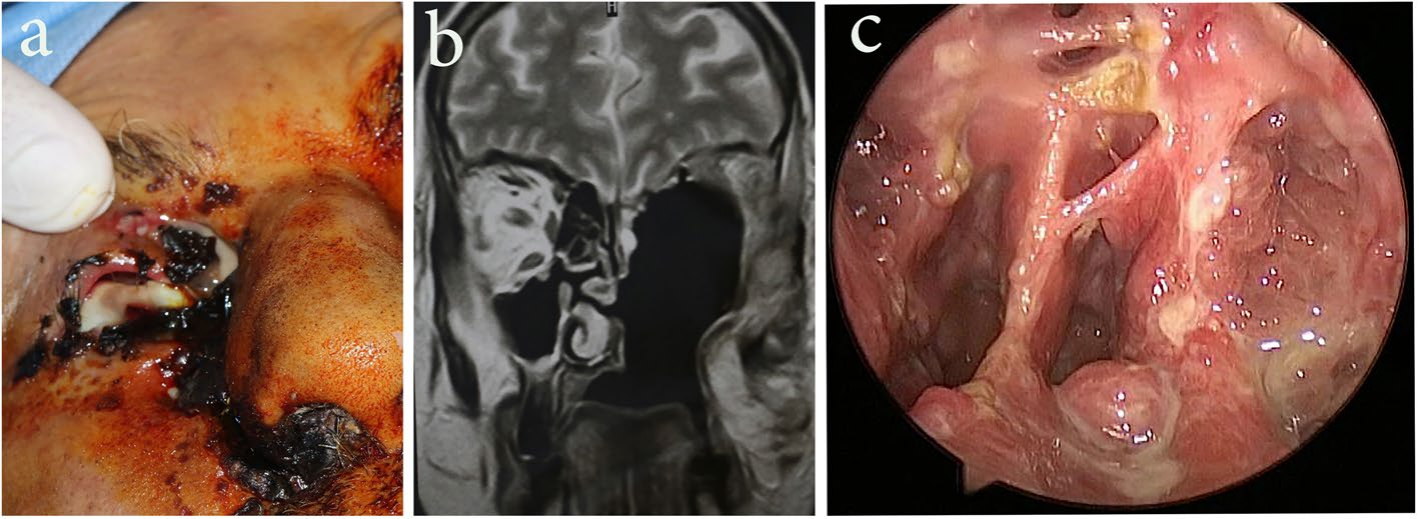
Three different cases. **a** Right eviscerated globe with areas of skin necrosis. **b** Three-month postoperative follow-up MRI nose and paranasal sinuses, showing left total maxillectomy and orbital exenteration cavity. **c** Post-operative endoscopic view after 3 months

Orbital exenteration was performed if the patient had a non-functioning eye (total visual loss and total ophthalmoplegia) (Fig. 1a) that was documented by an ophthalmologist, illustrated in imaging studies (especially MRI) by fungal invasion of the orbit, and proved pathologically from a previous biopsy or previous endonasal debridement. Maxillectomy was performed if clinically a necrosed hard palate, or imaging studies illustrated a destructed maxillary bone (total, subtotal, or inferior maxillectomy according to the extent of maxillary bone involvement).

Histopathological examination was performed under light microscopy, and fungal species were diagnosed by their morphology. Aseptate irregular 90° branching hyphae indicate Mucorales species, while aspergillus species have septate regular 45° branching hyphae [18].

### Follow-up

Patients were followed up every 15 days by endoscopic examination for 3 months post-operatively. A follow-up magnetic resonance imaging on the nose and paranasal sinuses was done after 3 months (Fig. 1b, c).

### Statistical analysis

Data were analyzed using IBM© SPSS© Statistics version 26 (IBM© Corp., Armonk, NY). Numerical variables are presented as mean and standard and inter-group differences are compared using the unpaired *t* test. Categorical variables are presented as numbers and percentages and differences are compared using the Pearson chi-squared test or Fisher’s exact test. Ordinal data are compared using the chi-squared test for trends. Receiver operating characteristic (ROC) curve analysis is used to examine the predictive value of continuous variables. Cox proportional hazards regression was used to examine the predictors of survival. *P* values < 0.05 are considered statistically significant.

## Results

Fifty-four patients (24 females, and 30 males) with a mean age of 48.1 years with a standard deviation of 16.5 (range 12–73 years). Fifty-two patients underwent surgical debridement (endoscopic, combined endoscopic, and external). Twenty-one patients died, so the mortality rate is 38.9% (Table 1). Age and sex had no impact on survival (*P* value 0.374 and 0.851, respectively) (Tables 1 and 2).

**Table 1:** Comparison of categorical variables in survivors and non-survivors

Variable	All patients (*n* = 54)	Survived (*n* = 33)	Died (*n* = 21)	*P* value
**Sex**				0.851†
*Females*	24 (44.4%)	15 (45.5%)	9 (42.9%)	
*Males*	30 (55.6%)	18 (54.5%)	12 (57.1%)	
**Comorbidity**				
*Chronic kidney disease*	10 (18.5%)	7 (21.2%)	3 (14.3%)	0.723‡
*Chronic liver disease*	2 (3.7%)	0 (0.0%)	2 (9.5%)	0.147‡
*Hematological malignancy*	2 (3.7%)	0 (0.0%)	2 (9.5%)	0.147‡
*History of thromboembolism*	9 (17.0%)	6 (18.2%)	3 (15.0%)	1.000‡
*Diabetes mellitus*	52 (96.3%)	33 (100.0%)	19 (90.5%)	0.147‡
**The onset of** ***diabetes mellitus***				0.694‡
*Newly diagnosed*	8 (15.4%)	6 (18.2%)	2 (10.5%)	
*Previously diagnosed*	44 (84.6%)	27 (81.8%)	17 (89.5%)	
**Glycemic control**				
*Diabetic ketoacidosis event*	34 (63.0%)	21 (63.6%)	13 (61.9%)	0.898†
*Poor glycemic control in hospital*	17 (31.5%)	9 (27.3%)	8 (38.1%)	0.404†
**COVID-19 PCR positivity**				0.653†
*Recent within 2 weeks*	20 (37.0%)	13 (39.4%)	7 (33.3%)	
*Previous within 2 months*	34 (63.0%)	20 (60.6%)	14 (66.7%)	
**Implicated pathogen**				0.329‡
*Mucoraceae*	42 (77.8%)	24 (72.7%)	18 (85.7%)	
Aspergillus	12 (22.2%)	9 (27.3%)	3 (14.3%)	
**Extent of disease**				
*Sinonasal tissue infarction*	54 (100.0%)	33 (100.0%)	21 (100.0%)	NA
*Skin infarction*	25 (46.3%)	15 (45.5%)	10 (47.6%)	0.876†
*Intraorbital extension*	38 (70.4%)	20 (60.6%)	18 (85.7%)	**0.049†**
*Intracranial extension*	22 (40.7%)	10 (30.3%)	12 (57.1%)	**0.050†**
**Severity of pulmonary affection**				0.796§
*No lung affection*	7 (13.0%)	5 (15.2%)	2 (9.5%)	
*Mild*	22 (40.7%)	13 (39.4%)	9 (42.9%)	
*Moderate*	7 (13.0%)	4 (12.1%)	3 (14.3%)	
*Severe*	18 (33.3%)	11 (33.3%)	7 (33.3%)	
**Debridement technique**	All debrided patients (*n* = 52)	Survived (*n* = 32)	Died (*n* = 20)	0.606†
*Endoscopic*	19/52 (36.5%)	10/32 (31.25%)	9/20 (45.0%)	
*Combined endoscopic and external*	33/52 (63.5%)	22/32 (68.75%)	11/20 (55.0%)	

Data are numbers or proportions (%). *n* number

*NA* = test not applicable

^†^Pearson chi-squared test

^‡^Fisher’s exact test

^‡^Chi-squared test for trend

**Table 2:** Comparison of numerical variables in survivors and non-survivors

Variable	All patients (*n* = 54)	Survived (*n* = 33)	Died (*n* = 21)	*P* value†
Age (years)	48.1 ± 16.5	49.7 ± 16.9	45.5 ± 16.0	0.374
FBG (mg/dl)	238.3 ± 76.7	246.7 ± 75.3	225.1 ± 78.9	0.319
2h-PPBG (mg/dl)	421.4 ± 107.1	415.2 ± 86.1	431.2 ± 135.6	0.598
HbA1c (%)	10.1 ± 2.0	10.2 ± 1.7	10.0 ± 2.5	0.742
SpO_2_ at diagnosis (%)	89.8 ± 5.9	89.8 ± 6.1	89.9 ± 5.8	0.982
Initial serum ferritin (ng/ml)	210.7 ± 102.8	189.7 ± 102.6	243.7 ± 96.2	**0.059**
LDH (IU/l)	464.8 ± 164.5	457.9 ± 167.0	475.5 ± 164.0	0.706
Initial CRP (mg/l)	63.1 ± 16.7	62.4 ± 16.9	64.2 ± 16.8	0.709

*FBG* fasting blood glucose, *2h-PPBG* 2 h post-prandial blood glucose, *HbA1c* glycosylated hemoglobin, *SpO*_*2*_ oxygen saturation, *LDH* lactate dehydrogenase, *CRP* C-reactive protein

Data are mean ± standard deviation

^†^Independent-samples *t* test

Besides being SARS-CoV-2 positive (20 patients) or recently recovered from SARS-CoV-2 infection (34 patients), most patients had comorbidities with diabetes mellitus being the most common comorbidity affecting 52 patients (96.3%), 10 had chronic kidney disease (18.5%), 2 had chronic liver disease (3.7%), 2 had leukemia (3.7%), and 9 had a history of thromboembolism (17.0%). The underlying medical condition had no predictive value for mortality (Table 1).

Assessment of the diabetes mellitus condition was thoroughly investigated, including onset of DM (8/52 were newly diagnosed), glycemic control (17/52 were poorly controlled), and diabetic ketoacidosis (34/52 had at least one event) (Table 1). Fasting blood glucose, two hours post-prandial blood glucose, glycosylated hemoglobin had a mean and standard deviation of 238.3 mg/dl ± 76.7, 421.4 mg/dl ± 107.1, 10.1% ± 2.0, respectively (Table 2). Both clinical and biochemical variables of DM have no impact in predicting mortality.

**Fig. 2:**
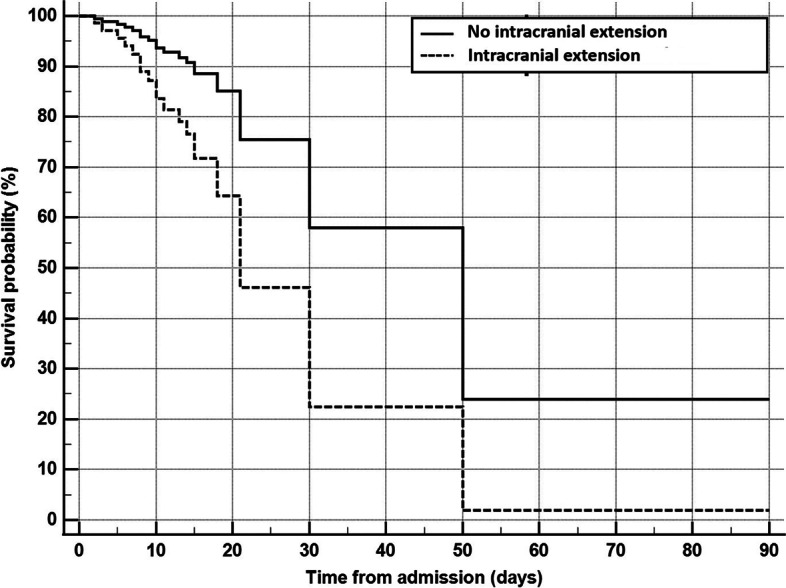
Cox proportional hazard survival curves for patients with or without intracranial extension. Intracranial extension is an independent predictor of mortality (Cox proportional hazard = 2.743, 95% CI = 1.046 to 7.199, *P* value = 0.040)

All patients had sinonasal tissue infarction. While the disease was confined to the sinonasal region in ten patients (18.5%), 25 patients (46.3%) had skin involvement, 38 patients (70.4%) had a visual loss, twenty-two patients had intracranial extension (40.7%). Intracranial extension and visual loss had a predictive value for mortality (P-value 0.050, and 0.049 respectively) (Table 2). However, by applying Cox proportional hazard regression, only intracranial extension is independent predictor of mortality (Cox proportional hazard = 2.743, 95% CI = 1.046 to 7.199, *P* value = 0.040) (Table 3 and Fig. 2).

**Table 3:** Cox proportional hazard regression for predictors of mortality in invasive fungal rhinosinusitis

Covariate	b	SE	Wald	*P* value	Proportional hazard	95% CI for proportional hazard
Intraorbital extension	1.263	0.753	2.813	0.094	3.535	0.808 to 15.466
Intracranial extension	1.009	0.492	4.204	**0.040**	2.743	1.046 to 7.199
Serum ferritin > 165 (μg/l)	2.531	0.721	12.333	**0.0004**	12.561	3.059 to 51.570

*b* = regression coefficient, *SE* = standard error, *95% CI* = 95% confidence interval

**Fig. 3:**
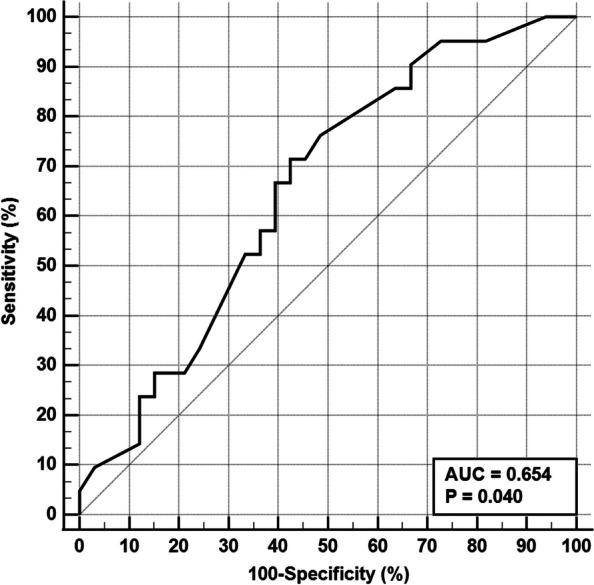
The receiver operating characteristic curve for prediction of mortality using serum ferritin. Serum ferritin > 165 ng/ml has fair predictive value with sensitivity of 71% and specificity of 58% (area under ROC curve = 0.654)

**Fig. 4:**
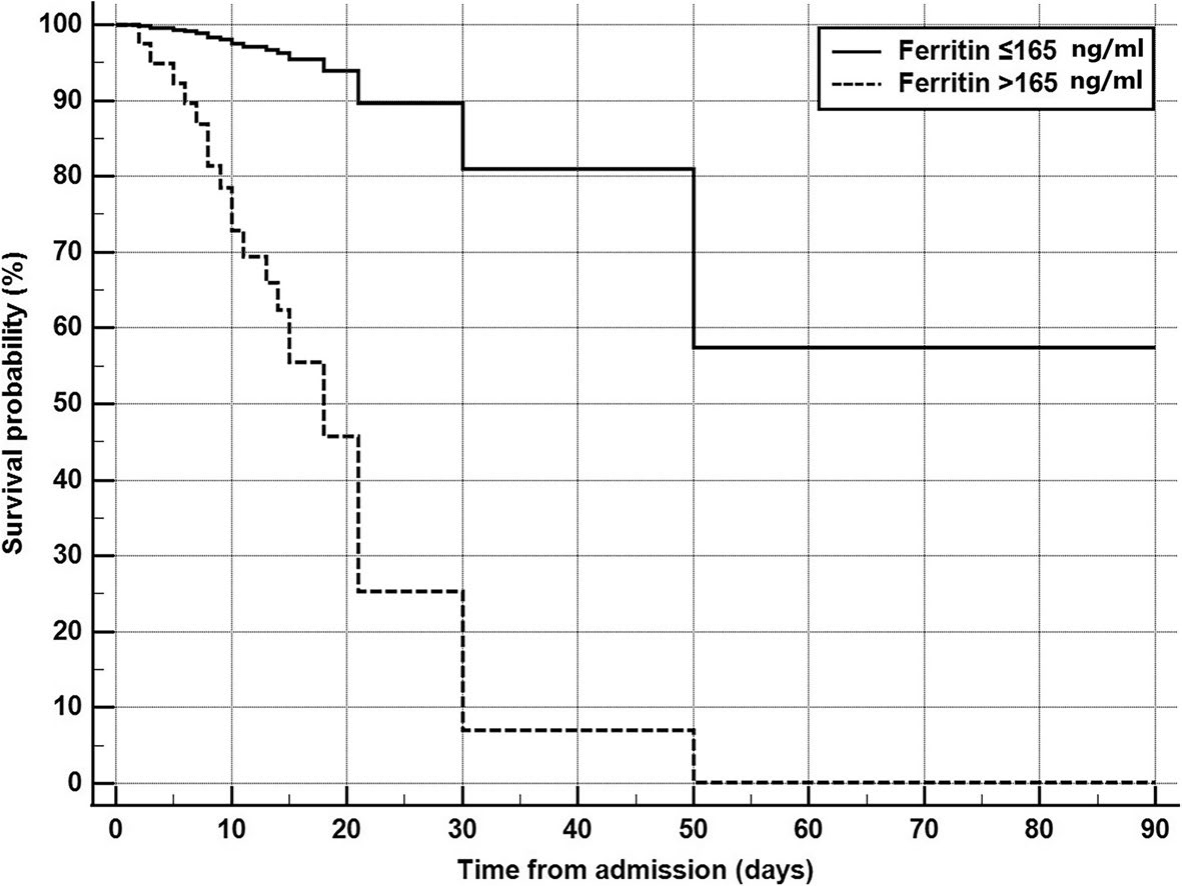
Cox proportional hazard survival curves for patients with serum ferritin > 165 ng/ml or ≤ 165 μg/l. Serum ferritin > 165 ng/ml is an independent predictor of mortality (Cox proportional hazard = 12.561, 95% CI = 3.059 to 51.570, *P* value = 0.0004)

Regarding other biochemical variables, serum ferritin, lactate dehydrogenase, and initial C-reactive protein had a mean and standard deviation of 210.7 μg/l ± 102.8, 464.8 IU/l ± 164.5, and 63.1 mg/l ± 16.7 (Table 2). Only serum ferritin level had a predictive value for mortality (*P* value 0.059), serum ferritin > 165 ng/ml has fair predictive value with a sensitivity of 71% and specificity of 58% (area under receiver operating characteristic “ROC” curve = 0.654) (Fig. 3). Also, serum ferritin > 165 ng/ml was independent predictor of mortality in patients with AIFR (Cox proportional hazard = 12.561, 95% CI = 3.059 to 51.570, *P* value = 0.0004) (Table 3 and Fig. 4).

Other variables were also compared between who survived and not, oxygen saturation at presentation (*P* value 0.982), SARS-CoV-2 positivity (within 2 weeks and 2 months) (*P* value 0.653), the severity of pulmonary affection (based on computed tomography of the chest) (*P* value 0.796), type of the invasive fungus (Mucorales and Aspergillus species) (*P* value 0.329), and debridement technique (endoscopic, or combined endoscopic and external) (*P* value 0.606). Those variables had no impact on survival.

## Discussion

AIFRS is an overwhelming infection that frequently occurs in severely immunocompromised patients, such as patients with uncontrolled diabetes mellitus, and hematological malignancies [19]. Recently, many reports of increasing incidence of AIFRS after or during SARS-CoV-2 infection have been published [8, 20, 21]. Since SARS-CoV-2 infected patients have elevated inflammatory cytokines and compromised cell-mediated immunity, as evidenced by lower counts of the cluster of differentiation 4 and 8 positive T-helper (CD4+ T and CD8+ T) cells, suggesting vulnerability to fungal co-infections [11].

The mortality rate in this study was 38.9%. This finding is consistent with those reported in the literature that ranged between 33% and 80% [22]. The wide range of the mortality rate might be due to differences in characteristics of the studied populations, the ability of complete debridement of the affected tissues, success in controlling the underlying predisposing factors, and the early initiation of treatment. Fifty-two patients (96.3%) in this study had surgery, which may result in a relatively lower mortality rate.

It was reported that the mortality rate is less in diabetics compared to non-diabetics [23]. In the present study, diabetes mellitus was the most common underlying comorbidity (52/54, 96.3%), which is much higher than in other previous reports (17–67%) [24].

This high incidence of diabetes mellitus in patients who have recent SARS-CoV-2 infection could be attributed to that SARS-CoV-2 is a diabetogenic infection that may cause altered glucose metabolism exacerbating preexisting diabetes or lead to new-onset diabetes and may lead to ketosis and ketoacidosis [25]. We had eight patients (8/52) with new-onset diabetes, moreover, 34 patients (34/52) experienced ketoacidosis.

Controlling the underlying etiology is imperative to outcomes, and DM is easier to control than other risk factors, such as hematologic malignancies, chronic kidney diseases, and chronic liver diseases. Therefore, the high proportion of diabetics in our study could have a favorable consequence on the outcome, while patients with chronic liver disease (2/54) and leukemia (2/54) represent a very low proportion (7.4%).

Patients who have impaired phagocytic function are at higher risk to develop AIFRS, as in normal conditions phagocytes can kill Mucorales by releasing oxidative metabolites, and defensins [26]. While in diabetic patients, elevated serum glucose weakens leukocyte activity (reduced chemotaxis and phagocytosis) [27], higher availability of glucose to Mucorales species, and decreased serum inhibitory action against Mucorales [28] leading to increased vulnerability for opportunistic infections. On the other hand, only controlling the serum PH and glucose level might not prevent disease progression, because the devitalized tissues are deprived of blood supply which results in localized acidosis [29], giving additional value for surgical debridement.

Monroe et al. reported that intracranial involvement and cranial neuropathy were associated with decreased survival [30]. On the contrary, intracranial or orbital involvement was not associated with a worse prognosis in the Gode et al. study [22].

Systemic antifungal alone has a poorer outcome than that if combined with surgical debridement [31]. Surgical debridement has been recognized as a chief component in the management of AIFRS, irrespective of the used approach [32], which can be endoscopic with good disease control [33]. Therefore, a combination of surgical debridement and systemic antifungal gives the best survival chance [34].

In our study, surgical intervention was done approximately within 48 from starting systemic antifungal therapy, together with controlling the underlying comorbidity. Endoscopic debridement was done for nineteen patients (36.5%) with confined involvement of the nasal cavity and paranasal sinuses. Thirty-three patients (63.5%) with extra-sinonasal spread had combined endoscopic and external open surgical debridement for orbital, palatal, and skin involvement. Surgical debridement reduces the fungal load, prevent fungal spread into dead tissues, enables systemic antifungal to diffuse more deeply into infected tissues, and allow post-operative endoscopic monitoring [35]. We did not find a difference in the mortality between endoscopic and combined techniques. This result is in line with reported by Kasapoglu et al. [33] that the survival rate of the patients who underwent open surgery (57.1%) was similar to that of patients treated endoscopically (47.3%). We believe that regardless of the approach whether open or endoscopic, debridement of all devitalized tissues until encountering bleeding margins is what matters.

In the current study, CRP level was not associated with increased mortality. While Gode et al. found that CRP level above 4 mg/dL was associated with increased mortality (area under the curve, 0.77; *p* 0.05, a sensitivity of 94.1%, and a specificity of 47.1%) [22]. Also, Cho et al. reported a slightly higher CRP level 5.50 mg/dL that was associated with increased mortality (area under the curve, 0.882; *p* 0.002) [36].

Ferritin is an inflammatory mediator that causes direct immune suppression, proinflammatory effects, and contributes to the cytokine storm [37]. Cytokine storm has been reported to cause fatal outcomes in SARS-CoV-2-infected patients. Moreover, increased ferritin levels were reported in patients with diabetes mellitus [38]. In our study, we found that serum ferritin level had a predictive value for mortality, serum ferritin > 165 ng/ml has a fair predictive value with a sensitivity of 71% and specificity of 58% (area under the curve = 0.654). Also, serum ferritin > 165 ng/ml was independent predictor of mortality in patients with AIFRS (Cox proportional hazard = 12.561, 95% CI = 3.059 to 51.570, *P* value = 0.0004). Likewise, Spellberg et al. [4] found that higher ferritin levels were associated with increased mortality. However, most patients who were included in their study with increased ferritin levels also had cancer. Therefore, the association between ferritin level and mortality could indicate increased baseline iron stores in those patients (malignancies, SARS-CoV-2 infection) resulting in more serious infection.

Both clinical and biochemical (fasting blood glucose, 2-h post-prandial blood glucose, glycosylated hemoglobin) variables of DM have no impact on predicting mortality. While Gür et al. [39] found that serum glucose level > 360 mg/dl had a poor outcome on survival for diabetic patients with AIFRS with an 83.3% sensitivity and specificity.

All patients in the study had sinonasal tissue involvement (100%), while intracranial extension, intraorbital involvement, and skin involvement were found in 40.7%, 70.4%, and 46.3% respectively. Intracranial extension and intraorbital involvement were found to be associated with a higher mortality rate (*P* value 0.050 and 0.049 respectively). However, only intracranial extension was an independent predictor of mortality (Cox proportional hazard = 2.743, 95% CI = 1.046 to 7.199, *P* value = 0.040). This supports previous studies concluding that intracranial and intraorbital involvement increased the mortality rate [33, 40–42]. The extensive extension is usually a result of delayed diagnosis, and usually is the main cause of mortality. After orbital involvement, the fungus can spread intracranially to the cavernous sinus, leading to cavernous sinus thrombosis [43, 44]. Then, internal carotid artery occlusion may occur resulting in coma and death [23].

Aspergillus was isolated in 12 patients, whereas Mucoraceae was isolated in 42 patients. Most of the study patients were diabetics (52/54), so Mucoraceae was the most commonly implicated pathogen. Kasapoglu et al. [33] reported that Mucoraceae was the main isolated fungi in their study. Ingley et al. [45] also observed a high prevalence of Mucoraceae within diabetics. Being infected with Aspergillus compared to Mucoraceae had no impact on mortality in our study, which is in line with Kasapoglu et al. and Valera et al. [46] but in contrary to Ingley et al. who reported that Mucoraceae had a higher mortality rate. Detection of the fungi subspecies could not be done, as fungal culture was not performed in this study, but Yohai et al. [23] reported that no survival difference was found between different Mucoraceae subspecies.

This study has several limitations: first, Amphotericin B was used for all the study populations so we could not assess a survival advantage with other newer systemic antifungals. Second, fungal culture was not available. Hence, we could not associate between different fungal species and mortality. Third, most of the patients were diabetics compared to a very low number of different underlying etiology such as malignancy (hematologic or non-hematologic).

Despite these limitations, the present study outlined factors apart from the treatment intervention (all patients had systemic antifungal therapy, and almost all patients underwent surgery), which were related to mortality in patients with SARS-CoV-2-related AIFRS. Our results suggest that patients presenting with intracranial extension or serum ferritin level above 165 ng/ml are more unlikely to survive.

## Conclusions

SARS-CoV-2-related acute invasive fungal rhinosinusitis has a mortality rate of 38.9%. Prognosis depends on the extent of the disease, with a higher mortality rate among those patients with intracranial extension, and patients with initial serum ferritin levels above 165 ng/ml

## Data Availability

The datasets used during the current study are available from the corresponding author on reasonable request.

## Abbreviations

AIFRS: Acute invasive fungal rhinosinusitis
DM: Diabetes mellitus
CD: Cluster of differentiation
rt-PCR: Reverse transcriptase-polymerase reaction
CT: Computed tomography
MRI: Magnetic resonance imaging
LDH: Lactate dehydrogenase
CRP: C-reactive protein
ROC curve: Receiver operating characteristic curve
